# P2A-Fluorophore Tagging of BRAF Tightly Links Expression to Fluorescence *In Vivo*

**DOI:** 10.1371/journal.pone.0157661

**Published:** 2016-06-27

**Authors:** J. Edward van Veen, Daphne R. Pringle, Martin McMahon

**Affiliations:** 1 Helen Diller Family Comprehensive Cancer, University of California San Francisco, San Francisco, CA, United States of America; 2 Huntsman Cancer Institute, University of Utah, Salt Lake City, UT, United States of America; Ohio State University Comprehensive Cancer Center, UNITED STATES

## Abstract

The *Braf* proto-oncogene is a key component of the mitogen-activated protein kinase signaling cascade and is a critical regulator of both normal development and tumorigenesis in a variety of tissues. In order to elucidate BRAF’s differing roles in varying cell types, it is important to understand both the pattern and timing of BRAF expression. Here we report the production of a mouse model that links the expression of *Braf* with the bright red fluorescent protein, tdTomato. We have utilized a P2A knock-in strategy, ensuring that BRAF and the fluorophore are expressed from the same endogenous promoter and from the same bicistronic mRNA transcript. This mouse model (*Braf*^*TOM*^) shows bright red fluorescence in organs and cell types known to be sensitive to BRAF perturbation. We further show that on a cell-by-cell basis, fluorescence correlates with BRAF protein levels. Finally, we extend the utility of this mouse by demonstrating that the remnant P2A fragment attached to BRAF acts as a suitable epitope for immunoprecipitation and biochemical characterization of BRAF *in vivo*.

## Introduction

BRAF plays a central role in the mitogen-activated protein kinase (MAPK) pathway, making it a key molecule for initiating cellular proliferation and differentiation in both normal cellular function and disease settings. Complete ablation of the *Braf* gene in mice is lethal by embryonic day (E) 12.5, with knockout (KO) embryos exhibiting vascular defects and general endothelial cell aberrations [1]. Further enforcing the important roles of BRAF in both vascular and cutaneous cell differentiation, germline mutations in *BRAF* can result in one of several genetic disorders with overlapping phenotypes that include cardiac defects and skin pathologies: Noonan; Costello; and cardio-facial-cutaneous syndromes [2]. These patients usually also present with neurologic and cognitive deficiencies, and, in the mouse, BRAF is necessary for the survival of motor and sensory neurons [3]. In humans, mutations in *BRAF*, most notably the BRAF^V600E^ mutation, are highly associated with a large number of cancers, e.g. 60% of melanomas harbor *BRAF* alterations [4]. Mutations in *BRAF* have also emerged as a key actionable mutation in non-small cell lung cancer (NSCLC) [5], and several clinical trials are examining the efficacy of BRAF blockade in *BRAF*^*mut*^ NSCLC patients.

Although KO of *Braf* and expression of the oncogenic BRAF^V600E^ has been modeled in the mouse, as of the writing of this manuscript, a reporter mouse for the expression of wild-type (WT) BRAF has not been generated [1,6]. Here we report the production of a BRAF-P2A-tdTomato (*Braf*^*TOM*^) mouse model. We show that the *Braf*^*TOM*^ mouse expresses bright tdTomato in several organs and cell types known to rely on BRAF signaling for normal functions, including the brain, hair follicles, and lungs. Additionally, the remnant P2A tag on BRAF allows for specific immunoprecipitation of BRAF, presenting a unique tool for the study of BRAF’s biochemical partners and interactions. Because of its vital roles in development and disease, we propose that this mouse will be a useful tool for the study of BRAF function *in vivo*.

## Methods

### Animal care and use

All mouse work was done with the approval of the University of California IACUC under approval #AN089594. Mice were housed in microisolator cages on ventilated racks in an AAALAC accredited facility. Mice were housed in groups, as possible, and were provided bedding enrichment. Animals were provided either standard laboratory rodent chow or Caphecci’s breeder diet when appropriate. Cages were supplied with water via a lix-it system built into the housing rack or with a water bottle placed in the microisolator cage. Institute husbandry staff performed twice daily healthchecks. Euthanasia was performed via CO_2_ gas inhalation via a compressed gas source at a fill rate of 10–30% of chamber volume per minute (optimized for specific chamber).

### ES cell targeting and screening

A targeting construct was made using standard cloning protocols, comprised of a ~4.8kb homology arm containing the final *Braf* exon and it’s 5’ intron. The endogenous *Braf* stop codon was omitted, and an optimized P2A motif [7] was fused in-frame, followed by: the bright red fluorescent protein, tdTomato; the CAAX membrane-targeting domain of *Kras;* and a stop codon. This was followed by an FRT flanked PGK-Puro-pA resistance cassette, and finally, by a ~3.7kb 3’ homology arm. The CAAX membrane-targeting domain was included in order to specifically tag tdTomato to the membrane so that downstream fluorescent staining/imaging of other proteins would not be occluded by diffuse cytoplasmic tdTomato. The construct was linearized by restriction digest and electroporated into *Braf*^*CA/+*^ ES cells which were selected using puromycin [6]. Three 96-well plates of resistant clones were screened via PCR using Q5 polymerase. In frame targeting of the wild-type allele vas verified by examining positive clones for tdTomato fluorescence on a standard inverted light microscope. *Braf*^*CA/+*^ ES cells were used in an effort to generate two mouse models, the *Braf*^*TOM*^ mouse described here, as well as a mouse model which expresses tdTomato only in cells also expressing BRAF^V600E^; this mouse will be fully described in a separate publication (J.E. van Veen, unpublished data).

### Embryonic Fluorescence

Timed pregnancies were established using *Braf*^*TOM/+*^ male mice crossed to wild type females. Pregnant dams were euthanized and embryos of different developmental ages were harvested unfixed and examined on a standard upright fluorescence microscope for native fluorescence. Images were stitched manually in Adobe Photoshop. tdTomato expressing embryos were immediately discernable from wild type littermates using red fluorescence, and genotypes were also verified by PCR.

### Organ Fluorescence

Various organs were harvested from *Braf*^*TOM/+*^ animals and immediately frozen unfixed in OCT (TissueTek, 4583). 10 μm sections were made from organs and maintained at -80°C. Frozen slides were thawed and fixed for 10 minutes in 4% PFA in PBS and native fluorescence was examined on a standard upright fluorescence microscope. Images were stitched automatically using Zeiss Zen 2 software.

### FACs analyses/FACS Westerns

Various organs were taken from *Braf*^*TOM/+*^ animals and minced using fine scissors in a .25 mg/ml solution of Liberase (Roche). Organs were allowed to digest in a 37° water bath for 15 minutes before being dissociated by pipetting up and down with a 1000 μl pipette tip. Red blood cell lysis was performed by the addition of BD PharmLyse (555899) to 1x concentration and samples were incubated for an additional 10 minutes. Samples were passed through a 100 μm filter. Filters were rinsed with 9 mL of ice cold Hanks Balanced Salt Solution, and flow through was pelleted via centrifugation. Pellets were resuspended in 10 mL ice cold HBSS and passed through a 70 μm filter. Pellets were then resuspended in 4% PFA in PBS for 15 minutes on ice, then pelleted and resuspended in PBS or blocking buffer. Notably, other fixatives that we tested, such as BD cytofix/cytoperm (554722) and various lower concentrations of PFA did a relatively poor job of preserving native tdTomato fluorescence. Thus, we determined that a minimum concentration of 4% PFA was necessary for preserving native tdTomato signal for downstream analyses. For antibody staining, cells were first blocked by resuspension in PBS + .25% Triton X-100 (PBT) and 10% Normal Donkey Serum. Following blocking, samples were resuspended in PBT containing primary antibodies used at the following concentrations: Anti-BRAF (Abcam, ab33899) 1:25; Anti-DSRed (Clontech, 632496) 1:200. Cells were then pelleted by centrifugation, washed with PBT, and resuspended in PBT containing appropriate AlexaFluor 488 conjugated secondary antibodies (Life Technologies, A-11034) at a concentration of 1:500.

For western analysis, naturally occurring populations of *Braf* expressing cells emerged when native tdTomato fluorescence was plotted against FSC-A as a measure of cell size. 100,000 cells from each population, representing differing levels of tdTomato expression, were sorted and pelleted on a minicentrifuge at 2000 RPM for five minutes. Liquid was carefully aspirated using a fine point pipette tip and cells were resuspended in 4X Laemmli’s sample reducing buffer. Samples were then run on a 4–12% Bis-Tris gradient gel (Life Technologies, NP0335) and transferred to a PVDF membrane via a dry transfer system (Life Technologies, IB21001 and IB24001). Antibodies used for blotting and their concentrations were as follows: BRAF (Santa Cruz Biotechnology, sc5284),1:500; pERK (Cell Signaling Technology, 4370), 1:1000; and b-Actin (Sigma, A5316), 1:5000. Fluorescent secondary antibodies of the appropriate species were used at a dilution of 1:6000 (LiCor, 926–32212 and 926–32213); proteins were visualized and bands quantified on a LiCor Odyssey. Band quantifications shown represent fluorescent signal from the bands shown, and are representative of multiple runs.

### Immunoprecipitation

Protein preparation: Snap-frozen brains and lungs were homogenized on ice in a glass tissue homogenizer containing immunoprecipitation lysis buffer (1% NP-40, 10% glycerol, 20 mM Tris pH 8, 137 mM Sodium Chloride, 1 mM Magnesium Chloride, 0.5 mM EDTA, 10 mM Sodium Pyrophosphate) supplemented with Halt Protease and Phosphatase Inhibitor Cocktail (Life Technologies, 78446). After homogenization, samples were lysed on ice for one hour and centrifuged at 14,000 rpm and 4°C for 10 minutes to pellet tissue debris. Protein concentrations were determined using Pierce BCA Assay Kit (Life Technologies, 23225).

Immunoprecipitation and Western Blot: 50 μg of protein at 0.2 μg/mL in IP lysis buffer containing protease inhibitor was cleared with 0.1 mg of normal rabbit IgG and 20 μL of a protein A agarose bead slurry (Cell Signaling Technologies, 9863) for 1 hour at 4°C with agitation. Beads were pelleted by centrifugation at 2500 RPM and 4°C for 30 seconds and cleared lysates were collected and transferred to a fresh tube. 5 μL anti-2A antibody was added (Millipore, ABS31) to cleared lysates followed by incubation overnight at 4°C with agitation. 20 μL of protein A agarose bead slurry was added and samples were incubated for another four hours at 4°C with agitation. Beads were pelleted as before and then washed thrice with 250 μL of cold IP lysis buffer containing protease inhibitor. Following final wash, 20 μL 4X Lammelli’s sample reducing buffer was added and samples were incubated for five minutes at 95°C (50 μg of input protein was treated similarly). Samples were then run on a 4–12% Bis-Tris gradient gel (Life Technologies, NP0335) and transferred to a PVDF membrane via a dry transfer system (Life Technologies, IB21001 and IB24001). Antibodies used for blotting and their concentrations were as follows: BRAF (Santa Cruz Biotechnology, sc5284), 1:500; CRAF (Cell Signaling Technology, 53745), 1:1000; and b-Actin (Sigma, A5316), 1:5000. Fluorescent secondary antibodies of the appropriate species were used at a dilution of 1:6000 (LiCor, 926–32212 and 926–32213) and proteins were visualized and bands quantified on a LiCor Odyssey. Band quantifications shown represent fluorescent signal from the bands shown, and are representative of multiple runs.

## Results and Discussion

### ES cell screening

**Fig 1:**
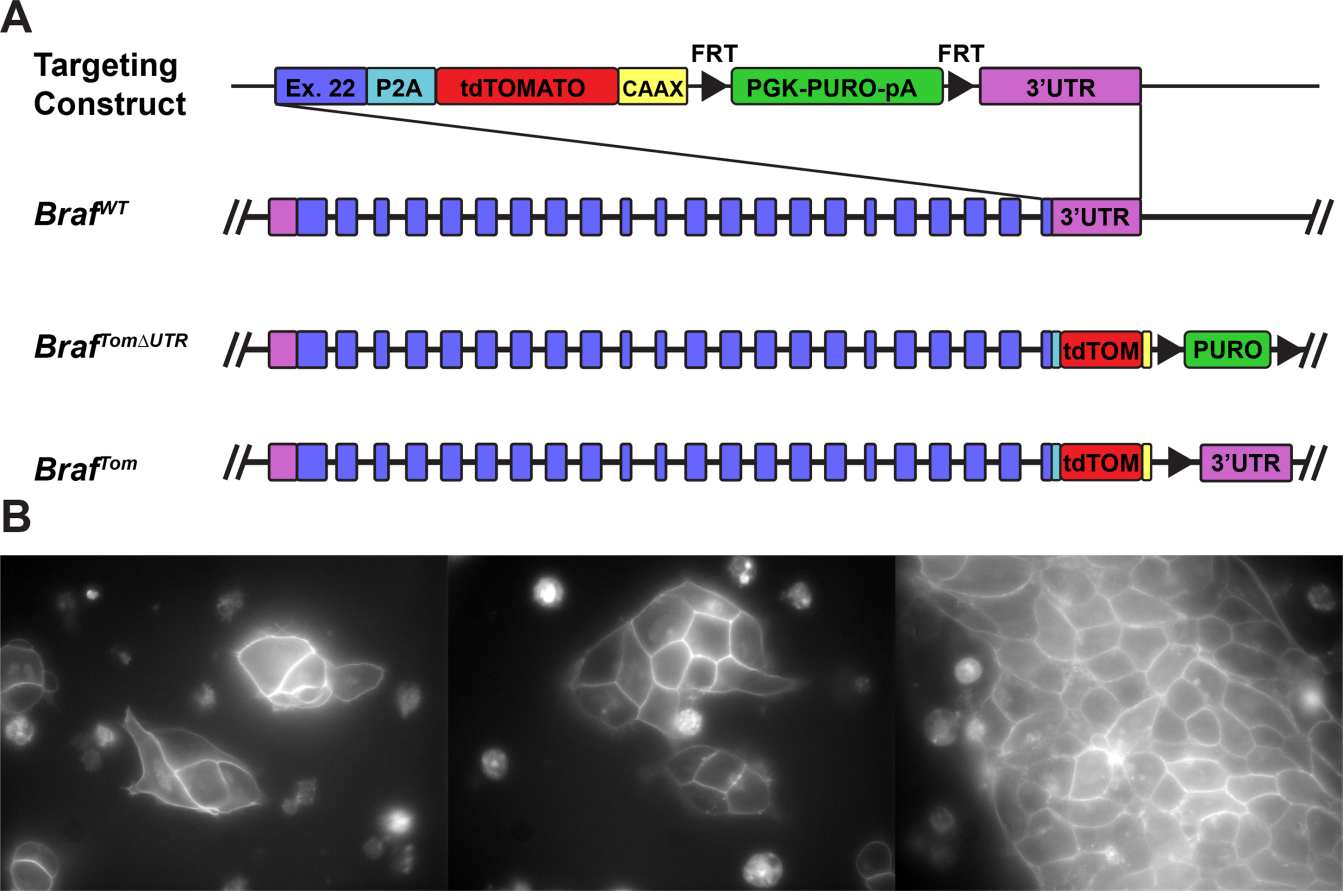
*Braf*^*TOM*^ ES cells exhibit membrane fluorescence. Shown is the structure (A) of the *Braf*^*TOM*^ targeting construct that fuses an in-frame P2A, tdTomato, and KRAS CAAX motif to the 3’ end of wild-type *Braf*. Successful targeting of the *Braf* locus results in the production of both BRAF and membrane targeted tdTomato as two separate proteins. Successfully targeted embryonic stem cells (B) demonstrate membrane-associated fluorescence confirming in-frame fusion in the 3’end of the *Braf* locus and validating functional components of the system. Scale bars 10 μm.

288 puromycin resistant ES cell colonies were screened via PCR for integration into the 3’end of the wild-type *Braf* locus. Of these, 22 showed a band corresponding to the predicted size for a homologous recombination event (data not shown). This recombination efficiency of 7.6% was similar to the targeting efficiency achieved in a previous attempt to target the *Braf* locus [6]. Positive colonies were expanded in vitro and examined for the presence of red fluorescence (Fig 1B). Roughly 50% (X/Y) of the ES cell colonies examined showed clear membrane associated fluorescence. As we targeted an ES cell line heterozygous for a conditional allele of *Braf* that halts transcription prior to the 3’ end of the locus (*Braf*^CA/+^), we expected only half of ES cell colonies to display fluorescence. We chose to use a picornavirus derived “self-cleaving” 2A sequence (P2A) to ensure that BRAF and tdTomato manifest as separate proteins. Unlike internal ribosome entry site (IRES) elements (6), 2A peptides are thought to result from ribosomal skipping, and thus there is no mechanism for a P2A peptide to display cryptic promoter activity, strongly implying that the presence of red fluorescence is indicative of in-frame fusion in the locus. Indeed, all fluorescent clones tested (n = 4) were, in fact, the result of recombination in 3’ end of the *Braf* locus.

### Embryonic expression of BRAF

**Fig 2:**
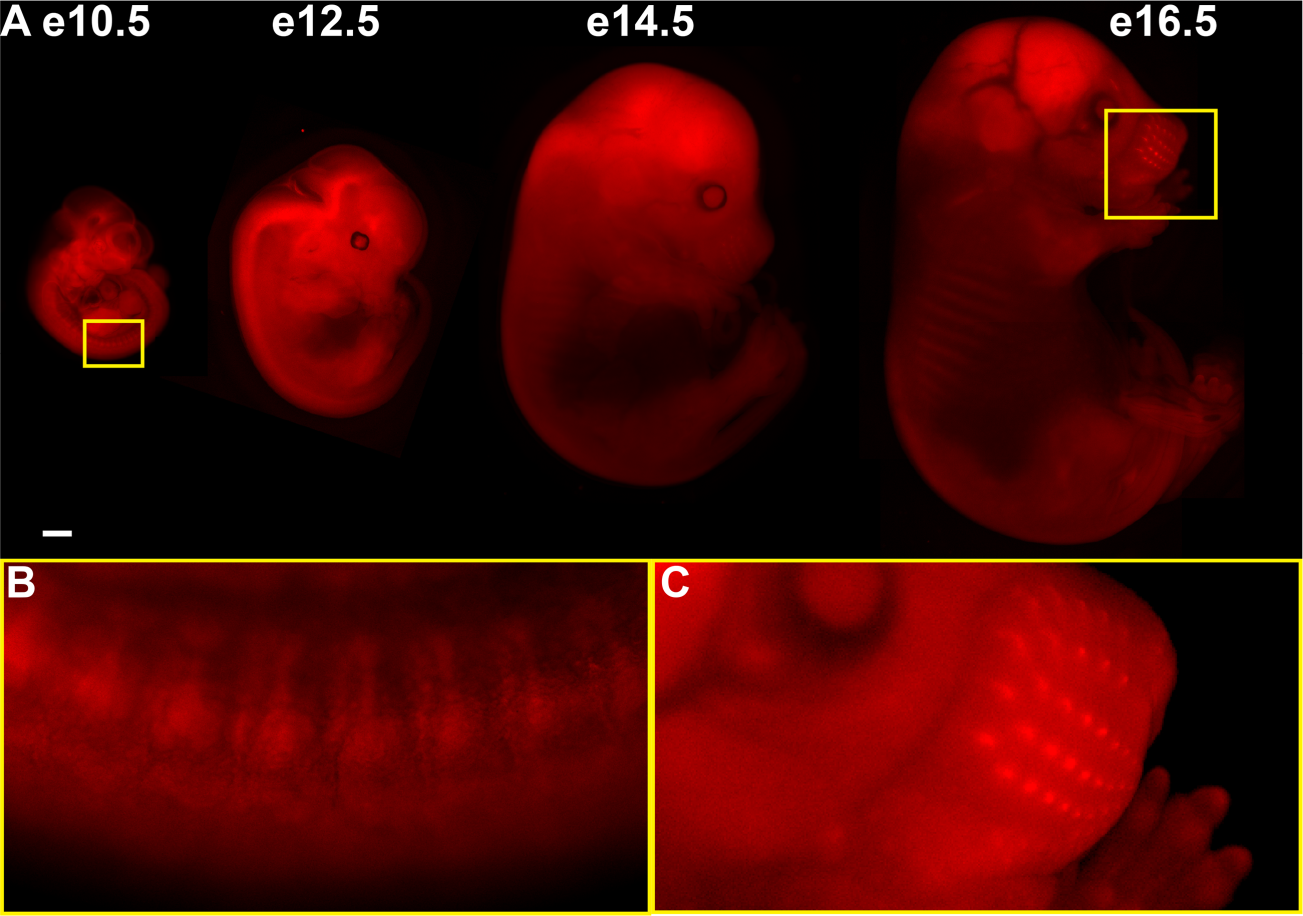
tdTomato fluorescence highlights BRAF expression in embryonic structures. Native fluorescence (A) from various developmental stages of *Braf*^*TOM/+*^ embryos. (B) BRAF expression in dorsal root ganglia (DRGs). (C) BRAF expression in the hair follicles of the developing mouse snout. E12.5, E14.5, and E16.5 embryos demonstrate high levels of BRAF expression in the developing brain. Scale bars 1 mm.

Embryos of different ages were examined for the presence of red fluorescence. As expected if inheritance of the *Braf*^*TOM*^ allele is Mendelian, roughly 50% of embryos derived from a *Braf*^*TOM/+*^ x WT cross displayed strong red fluorescence (Fig 2A). Of note, *Braf*^*TOM/TOM*^ animals were also generated at Mendelian frequencies and were viable, fertile, and healthy (data not shown). Non-fluorescent littermates showed negligible or undetectable signal in the red channel when captured and displayed with the same exposure and scaling settings (data not shown). All genotypes were confirmed via PCR, and 100% of *Braf*^*TOM/+*^ animals exhibited fluorescence. At E10.5 red fluorescence is widespread throughout the embryo. We observed a repeating pattern in the spinal cord, which upon closer inspection appears to be dorsal root ganglia (DRGs) (Fig 2A, E10.5, yellow inset). Strong expression of *Braf* in the developing DRGs is unsurprising as it has previously been shown to be a critical mediator of survival in response to neurotrophic factors in motor and sensory neurons of the peripheral nervous system [3]. As development proceeds, tdTomato fluorescence becomes most striking in the CNS, first appearing in the pons (E12.5) and finally in the brain (E14.5-E16.5) (Fig 2B). Further studies will be required to determine the precise location and function of *Braf* expression in these structures. Notably, by E16.5, intense tdTomato expression is visible in the hair follicles in the snout (E16.5, yellow inset). Again, this correlates with known roles of *Braf* in development; MAPK signaling has been repeatedly demonstrated to be critical in hair development and homeostasis as mutations in the pathway can lead to progressive alopecia and premature follicle differentiation [8,9].

### Expression of BRAF in the adult brain and lung

**Fig 3:**
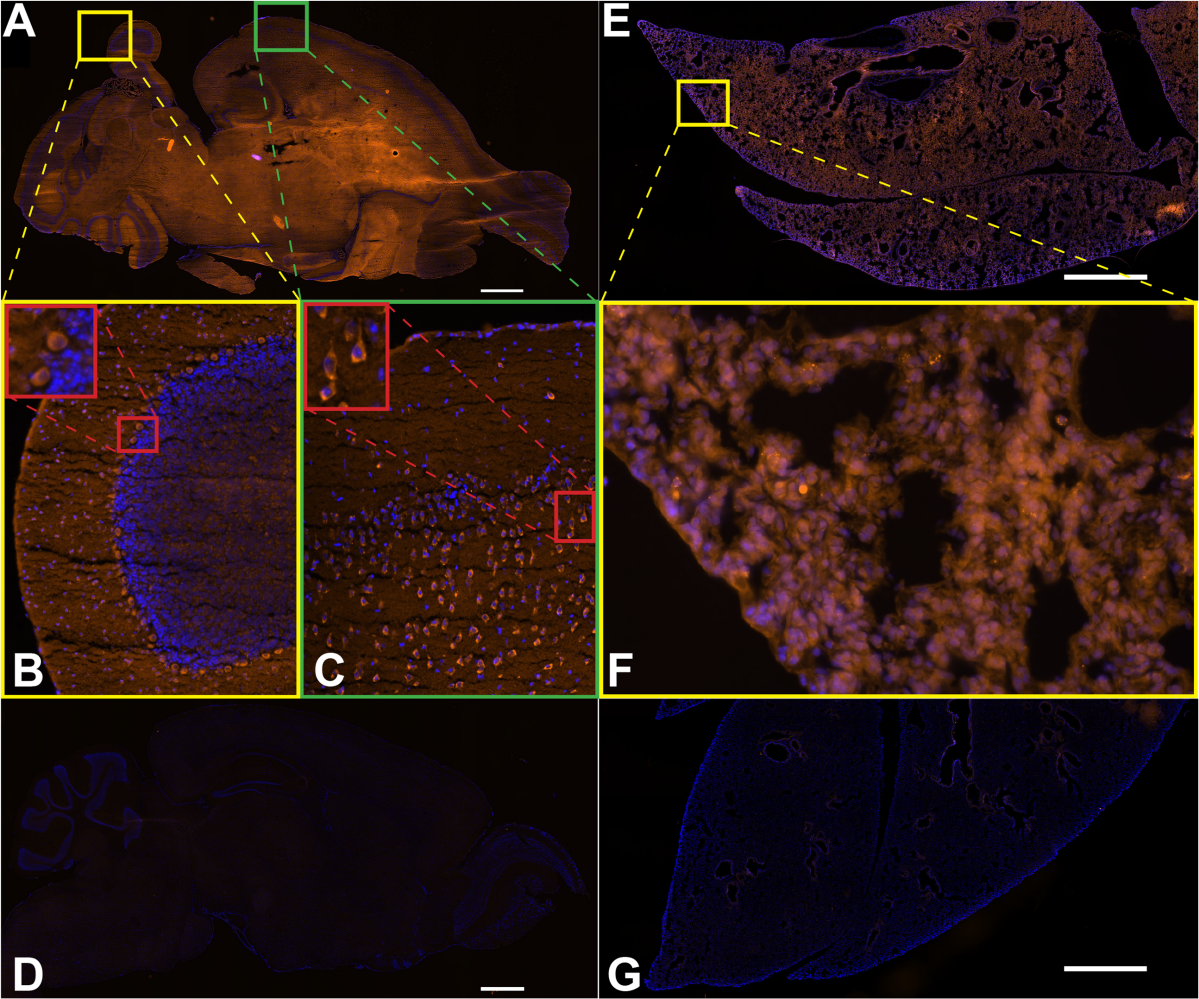
Adult cell types sensitive to BRAF perturbation show strong tdTomato fluorescence. Native fluorescence (red) and DAPI (blue) in adult *Braf*^*TOM/+*^ brain and lung structures. (A) 10 μm frozen sections taken from *Braf*^*TOM/+*^ animals show widespread fluorescence in the brain. (B) A region of the cerebellum illuminates BRAF expression within Purkinje neurons. (C) A region of the cortex demonstrating a specific BRAF expression pattern. Red inset shows what appear to be cortical neurons with a high level of BRAF expression. (D) Brains of wild-type littermates sectioned and imaged side-by-side under the same conditions show negligible autofluorescence by comparison. (E) 10 μm frozen sections taken from *Braf*^*TOM/+*^ animals show widespread fluorescence in the lung. (F) a region of the distal lung showing tdTomato fluorescence, though no individual cell types are immediately apparent. (G) As in the brain, lungs from wild-type littermates sectioned and imaged under the same conditions show comparatively negligible autofluorescence. Scale bars 100 μm.

To examine in more detail, the cells from organs where BRAF/MAPK signaling is known to be highly important, we made sections from fresh frozen brains and lungs taken from *Braf*^*TOM/+*^ adult animals. *Braf*^*TOM/+*^ brains demonstrate widespread but not ubiquitous red fluorescence (Fig 3A). Increased magnification of the cerebellum (Fig 3B) shows bright fluorescence in the Purkinje neurons. Further magnification demonstrates that neuronal morphology is readily assayable (Fig 3B, red inset), making the *Braf*^*TOM/+*^ mouse a potential tool for studying the effects mutations or drugs have on neuronal homeostasis *in vivo*. Once again, the expression pattern of tdTomato is in accordance with known roles of *Braf*, as expression of *Braf* in the cerebellum is reportedly highest in Purkinje neurons [10] and developmental ablation of *Braf* leads to reduced number and ectopic placement of Purkinje neurons [11]. A subset of cells in the cortex also display discrete red fluorescence (Fig 3C). Closer examination (Fig 3C, red inset) reveals a neuronal like morphology, with leading process-like structures. Again these patterns are concordant with the finding that MAPK signaling is crucial in the specification of neurons in the cortex [12]. Together, these distinct expression patterns in the brain suggest the *Braf*^*TOM*^ mouse could be utilized in identifying key processes in the brain that require BRAF, that may be disrupted in patients with neurological defects caused by germline *BRAF* mutation.

Importantly, these patterns of fluorescence are not the result of autofluorescence, as wild-type littermate brains display essentially absent signal when captured with precisely the same exposure settings (Fig 3D). We also examined tdTomato fluorescence in the lung, and while *Braf*^*TOM/+*^ animals displayed widespread signal (Fig 3E) and littermate controls displayed essentially absent signal (Fig 3G), no particular cell types or structures were immediately apparent (Fig 3F), suggesting BRAF expression in the lung is ubiquitous, which is supported by data from the Human Protein Atlas [13].

### FACS analysis of correlation of BRAF and tdTomato expression

**Fig 4:**
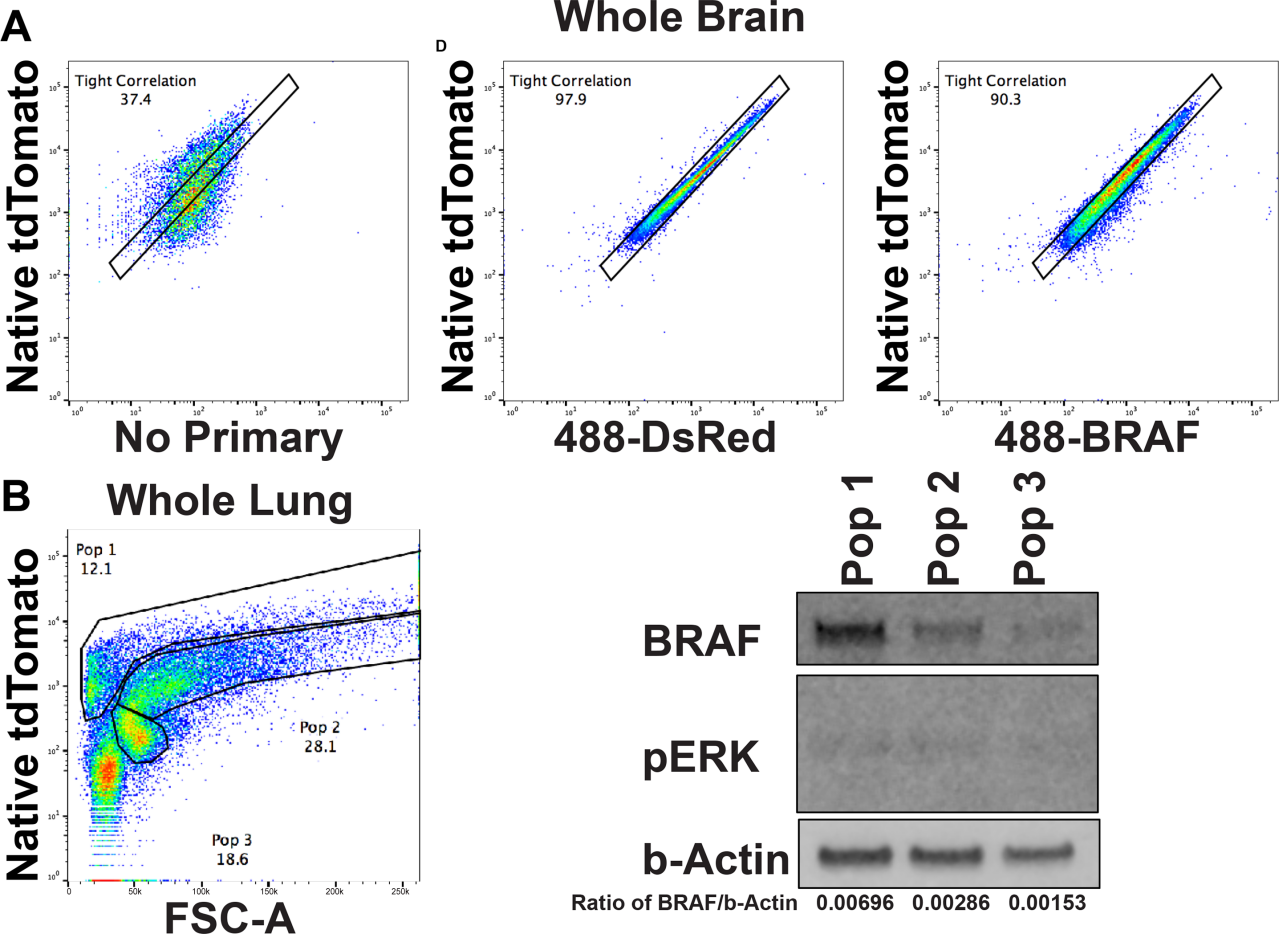
FACS analysis of the correlation between BRAF expression and native tdTomato fluorescence. (A) Dissociated *Braf*^*TOM/+*^ brains antibody stained for markers of interest (X axes) and compared to level of red fluorescence. Alexa 488 secondary antibody alone showed negligible correlation with red fluorescence. In contrast, a primary antibody that recognizes tdTomato shows a very tight correlation with tdTomato fluorescence. Staining with a BRAF reactive primary antibody also shows very good correlation with tdTomato fluorescence. (B) *Braf*^*TOM/+*^ analyzed for tdTomato fluorescence versus a surrogate for size (FSC-A) fell into three major natural populations of cells. Population 1, which had the highest level of tdTomato intensity per area showed the most BRAF immunoreactivity on western blot. Population 2, which showed less tdTomato intensity, also showed decreased BRAF immunoreactivity. Population 3 showed the weakest tdTomato intensity and BRAF immunoreactivity. Quantification of the ratio of BRAF to β-actin in each population is also shown. Similar results were seen with cells sorted from the lungs of *Braf*^*TOM/+*^ mice (data not shown).

The utility of any mouse model depends largely upon how faithfully the surrogate reporter recapitulates expression of the gene of interest. As most levels of gene regulation are maintained using our approach, it seems reasonable that the levels of tdTomato and BRAF would largely mirror each other. To demonstrate directly that the level of tdTomato fluorescence we observe varies in a linear fashion as BRAF protein levels vary, we utilized an immuno-FACS based analysis. Dissociated brains from *Braf*^*TOM/+*^ mice were fixed in a manner that largely preserved native tdTomato fluorescence. tdTomato immunoreactivity showed a remarkably linear relationship between green and red fluorescence (Fig 4A), whereas omitting primary antibody from the staining resulted in a relatively poor correlation. BRAF immunoreactivity was also highly correlated to native red fluorescence, albeit not as well as what we observed with anti-tdTomato staining. Because this type of FACS analysis analyzes cells on a one-by-one basis, we were able to observe that >90% of individual cells showed a very good correlation between tdTomato fluorescence and BRAF expression. Interestingly, of those cells that did not show a strong correlation between BRAF and tdTomato levels, the majority apparently had higher levels of BRAF than tdTomato. There are many possible explanations for such an observation. The CAAX membrane targeting domain on tdTomato could potentially shorten its protein stability or half-life, although the CAAX tag has been found to not influence the stability of other fluorophores [14], the potential remains, and may need to be explored further. It is also possible that the antibody staining is the more accurate measure, and that in some cells, post-translational events stabilize BRAF protein, leading to lower red fluorescence than what would best correlate with BRAF protein levels. Additionally, some antibody cross reactivity with other RAF family isoforms or other non-specific binding could lead to spuriously high antibody staining, in which case tdTomato expression would be more accurate for quantification of BRAF protein levels.

In order to overcome these caveats, we utilized a FACs-to-Western blot strategy; Western blot analysis is less prone to artifacts resulting from non-specific antibody binding allowing molecular weight to be a criterion by which to discern specificity. To make use of a Western blot in this case, we analyzed dissociated brains from *Braf*^*TOM/+*^ animals to copmare red fluorescence to forward scatter (FSC-A) as a surrogate for cell size. Three major populations of cells naturally emerged (Fig 4B). 100,000 cells from each population were sorted, lysed, and analyzed. As expected, there was a direct correlation between red fluorescence and BRAF protein levels, which was confirmed by quantification of fluorescent signal from the secondary antibody on Western blot (Fig 4B). Along with the observed patterns of fluorescence in cells expected to have high levels of BRAF, these data argue strongly that P2A-fluorophore tagging of an endogenous protein results in very faithful recapitulation of gene expression by red signal.

### Immunoprecipitation of BRAF using anti-2A antibody

**Fig 5:**
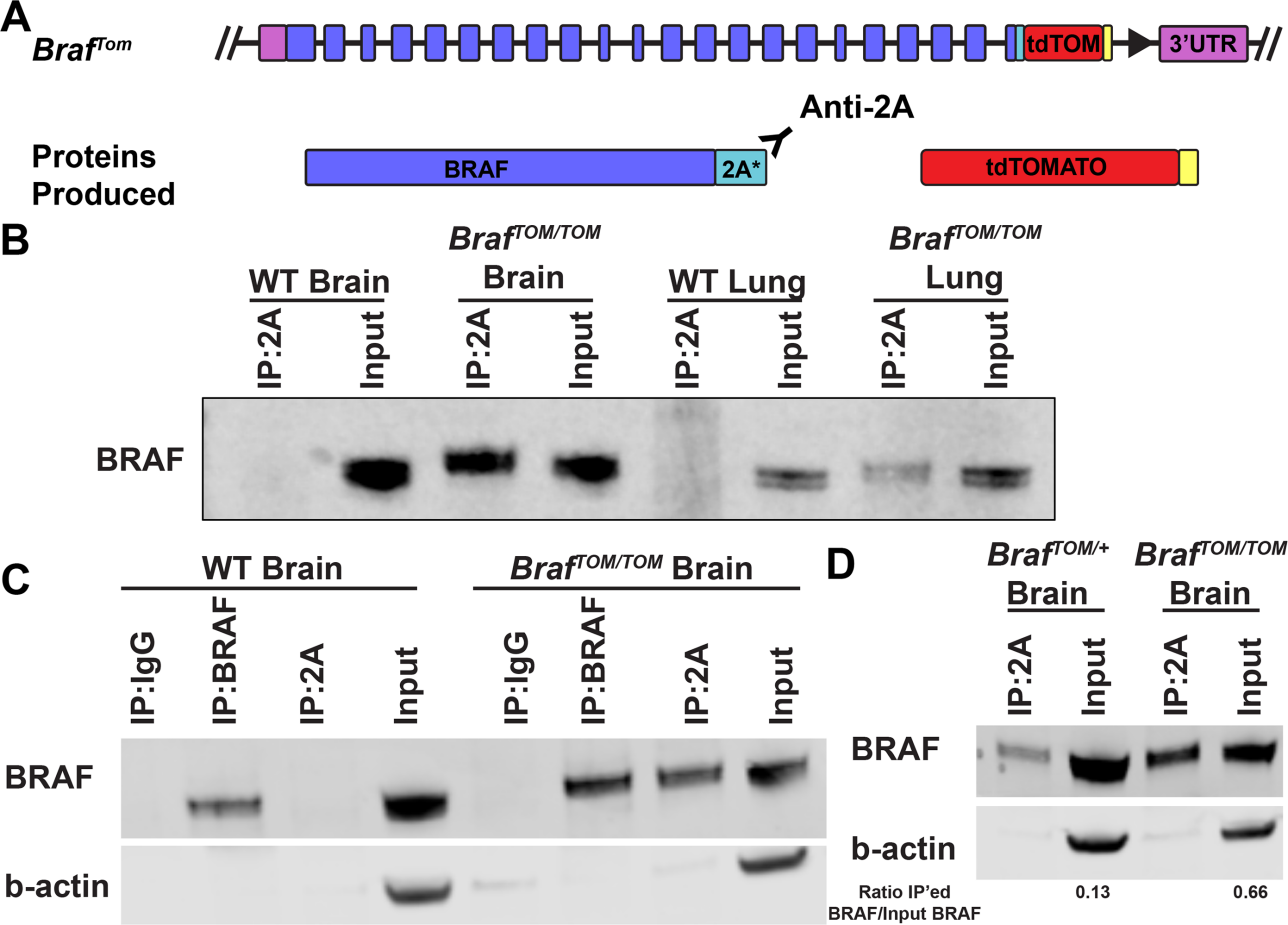
Epitope utility of the cleaved P2A peptide. (A) Cleavage of the P2A peptide leaves behind a 21 amino acid tail on the N-terminal protein, which may serve as an epitope for antibody mediated procedures. (B) The anti-2A antibody is used to efficiently immunoprecipitate BRAF protein from *Braf*^*TOM/TOM*^ brain and lung lysates but does not show any detectable non-specific binding of untagged BRAF protein in wild-type brain or lung lysates. (C) The anti-2A and anti-BRAF antibodies show similar efficacy in immunoprecipitation of BRAF protein from *Braf*^*TOM/TOM*^ brain lysates, but only the anti-BRAF antibody is able to immunoprecipitate BRAF from wild-type lysates. (D) IP with the anti-2A antibody quantitatively distinguishes between *Braf*^*TOM/+*^ and *Braf*^*TOM/TOM*^ lysates. Quantification of the ratio of IP’ed BRAF to input BRAF is shown.

Cleavage of the P2A motif leaves behind a 21 amino acid remnant on the N-terminal protein (Fig 5A) [15]. At time of writing, there exists one commercially available antibody reported to recognize this sequence. To test the utility of the P2A tail as an epitope for further biochemical analyses, we immunoprecipitated (IP) protein from lysates derived from *Braf*^*TOM/TOM*^ brains and lungs along with their wild-type littermates. Brain and lung were chosen as tissues to explore biochemical characterization due to ease in generating high protein concentration lysates and known critical functions of BRAF in both tissues. When analyzed by western blot, it became clear that we were able to specifically IP BRAF from *Braf*^*TOM/TOM*^ brain and lung lysates, and we did not observe any BRAF protein in the IPs performed on littermate controls (Fig 5B). To examine the efficiency of this immunoprecipitation, we directly compared IPs performed with either anti-2A or anti-BRAF on either wild-type or *Braf*^*TOM/TOM*^ brain lysates. In *Braf*^*TOM/TOM*^ lysates, a similar amount of BRAF protein was IP'ed using either anti-2A or anti-BRAF (Fig 5C). Again demonstrating specificity, anti-BRAF IP'ed similar amounts of BRAF protein from either *Braf*^*TOM/TOM*^ or wild-type brains, whereas anti-2A only IP'ed detectable levels of BRAF protein from *Braf*^*TOM/TOM*^ lysates (Fig 5C). We also examined the ability of IP with the anti-2A antibody to distinguish between *Braf*^*TOM/+*^ and *Braf*^*TOM/TOM*^ lysates as anti-2A IP should pull-down ~50% of all endogenous BRAF in the *Braf*^*TOM/+*^ animals and 100% of endogenous BRAF in *Braf*^*TOM/TOM*^ brain lysates. We show that while the efficiency is not perfect, quantification does show a greater than two-fold increase in IP’ed BRAF in *Braf*^*TOM/TOM*^ brain lysates compared to *Braf*^*TOM/+*^ lysates (Fig 5D). It is also important to note that in these and all other blots we have analyzed (n≥3), we have never observed a larger molecular weight band that would correspond to the combined size of BRAF and tdTomato, indicating that P2A cleavage is highly efficient in a large number of mouse tissues in vivo.

## Conclusions

Here we have described a novel model of wild-type *Braf* expression. Notably, specific expression patterns, correlating with known functions of BRAF, have been observed in embryos and adult animals, and are currently being investigated by our lab and collaborating labs. We have demonstrated for the first time that this P2A-fluorphore tagging approach results in faithful recapitulation by the fluorophore of the protein of interest. Furthermore, this model is ideal for use in further biochemical characterization, as the P2A remnant left on the N-terminal protein allows for specific immunoprecipitation by a commercially available antibody. IP with the anti-2A antibody results in efficient and reproducible isolation of BRAF in a dose-dependent manner from whole organ disassociations, making these *Braf*^*TOM*^ mice a useful new tool for the study of WT BRAF protein function on a cell by cell basis *in vivo*.

## Supporting Information

S1 FCS FCS raw data files (used in making Fig 4A). No primary antibody.
(FCS)

S2 FCS FCS raw data files (used in making Fig 4A). Anti-DSred antibody.
(FCS)

S3 FCS FCS raw data files (used in making Fig 4A). Anti-Braf antibody.
(FCS)

S4 FCS FCS raw data files (used in making Fig 4B). FCS sorting strategy for western.
(FCS)
